# 5‐ALA/SFC enhances HO‐1 expression through the MAPK/Nrf2 antioxidant pathway and attenuates murine tubular epithelial cell apoptosis

**DOI:** 10.1002/2211-5463.12729

**Published:** 2019-09-30

**Authors:** Chi Liu, Masayuki Fujino, Shuoji Zhu, Yoshitaka Isaka, Hidenori Ito, Kiwamu Takahashi, Motowo Nakajima, Tohru Tanaka, Ping Zhu, Xiao‐Kang Li

**Affiliations:** ^1^ Division of Transplantation Immunology National Research Institute for Child Health and Development Tokyo Japan; ^2^ AIDS Research Center National Institute of Infectious Diseases Tokyo Japan; ^3^ Guangdong Cardiovascular Institute Guangdong Academy of Medical Sciences Guangdong Provincial People's Hospital Guangzhou China; ^4^ Department of Nephrology Osaka University Graduate School of Medicine Japan; ^5^ SBI Pharmaceuticals Co., Ltd. Tokyo Japan

**Keywords:** 5‐aminolevulinic acid, apoptosis, Cyclosporine A, heme oxygenase‐1, oxidative stress

## Abstract

Cyclosporin A (CsA) is a common immunosuppressant, but its use is limited as it can cause chronic kidney injury. Oxidative stress and apoptosis play a key role in CsA‐induced nephrotoxicity. This study investigated the protective effect of 5‐aminolevulinic acid and iron (5‐ALA/SFC) on CsA‐induced injury in murine proximal tubular epithelial cells (mProx24). 5‐ALA/SFC significantly inhibited apoptosis in CsA‐treated mProx24 cells with increases in heme oxygenase (HO)‐1, nuclear factor E2‐related factor 2 (Nrf2), and p38, and Erk‐1/2 phosphorylation. Moreover, 5‐ALA/SFC suppressed production of reactive oxygen species in CsA‐exposed cells and inhibition of HO‐1 suppressed the protective effects of 5‐ALA/SFC. In summary, 5‐ALA/SFC may have potential for development into a treatment for the anti‐nephrotoxic/apoptotic effects of CsA.

**Abbreviations**
5‐ALA: 5‐aminolevulinic acid
ARE: antioxidant response elements
CM‐H2DCFDA: 5/6‐chloromethyl‐2′,7′‐dichlorodihydrofluorescein diacetateacetyl ester
CO: carbon monoxide
CsA: Cyclosporin A
HO: heme oxygenase
Keap1: Kelch‐like ECH‐related protein 1
Nrf2: nuclear factor E2‐related factor 2
PI: propidium iodide
ROS: reactive oxygen species
SFC: sodium ferrous citrate

Cyclosporine A (CsA) is one of the most effective and widely used immunosuppressants in organ transplantation. CsA is also used in the treatment of several autoimmune diseases. However, its clinical use is limited by the adverse effect of renal injury. In fact, CsA‐induced end‐stage renal failure is a major complication and an increasing problem in non‐renal solid organ transplant recipients. CsA‐induced nephropathy includes tubulointerstitial fibrosis, arteriolopathy, and tubular atrophy. Although the exact mechanism of CsA‐induced nephrotoxicity is unclear, numerous studies have suggested that CsA activates the intrarenal renin–angiotensin system and increases the expression of endothelin‐1, leading to afferent arteriolar vasoconstriction, a reduction in renal blood flow, oxidative stress, and eventually, apoptosis in the kidney 1. Whereas CsA increases hypoxia and free radical production in the kidney, some antioxidants are able to prevent CsA‐induced nephropathy 2. Previous studies have reported a multifactorial mechanism underlying CsA‐induced nephropathy. In practice, therefore, it is necessary to control inflammation, apoptosis, and fibrosis associated with oxidative stress to delay the progression of chronic CsA‐induced nephropathy. Doing so can allow patients to benefit from these useful antioxidant drugs while preventing serious complications.

The analysis of the renal protective responses and specific intracellular signaling molecules or gene activation pathways in CsA nephropathy has provided insights into the pathomechanism of the condition and possible treatments. Recently, the nuclear factor E2‐related factor 2 (Nrf2) was found to be a key transcription factor binding to the antioxidant response elements (ARE) present in the promoter regions of various antioxidants and phase II enzymes, which exert detoxification effects typically through conjugation, thereby inactivating potentially dangerous substrates, such as glutathione peroxidase, catalase, and superoxide dismutase, by increasing their solubility and facilitating their excretion 3. Nrf2 apparently functions by sensing cytoplasmic oxidative stress or specific chemical agents after it is released from its repressor, Kelch‐like ECH‐related protein 1 (Keap1) 4. Heme oxygenase (HO)‐1 is one of the Nrf2 target genes and catalyzes the metabolism of heme, carbon monoxide (CO), and bilirubin 5. The HO enzyme system catalyzes the rate‐limiting step in the degradation of heme. Two major HO isoforms, HO‐1 and HO‐2, which are the products of different genes, have been identified. HO‐1 is an inducible form of HO whose upregulation generates cytoprotective products, such as bilirubin and CO 6. HO‐1 is thought to exert its protective effects in the kidney by degrading heme, a pro‐oxidant, and producing anti‐inflammatory, antioxidant, and anti‐apoptotic metabolites 7.

5‐aminolevulinic acid (5‐ALA), commonly found in plants, bacteria, fungi, and animals, is a precursor to biosynthetic tetrapyrroles, including chlorophyll, vitamin B12, and heme 8. 5‐ALA is an endogenous amino acid in animals and is the first compound produced by 5‐ALA synthase in the heme biosynthetic pathway 8. Recent studies have shown that 5‐ALA has cardioprotective, antifibrotic, antitumor, anti‐inflammatory, and antioxidative effects 9, 10, 11. Furthermore, 5‐ALA is known to induce HO‐1 expression in kidney cells, both *in vivo* and *in vitro* 8, 12, 13. 5‐ALA has been shown to activate HO‐1 in mouse inflammatory disease models and inhibit the reduction of inflammatory responses such as TNF‐α and iNOS expression, but upregulate the anti‐inflammatory response 8, 13. Treatment with 5‐ALA also showed the induction of immune tolerance to long‐term graft receptivity 14.

The purpose of this study was to investigate the possible protective effect of the antioxidant and anti‐apoptotic activity of 5‐ALA against CsA‐induced injury in renal tubular epithelial cells (mProx24).

## Materials and methods

### Cell culture and treatment

A murine renal proximal tubular epithelial cell line (mProx24; provided by CMIC Group, Tokyo, Japan) 15 derived from C57BL/6J adult mouse kidney was maintained in Dulbecco's modified Eagle's medium (supplemented with 1000 mg·L^−1^ d‐glucose, 10% FBS, 100 U·mL^−1^ penicillin, and 100 mg·mL^−1^ streptomycin) at 37 °C in 5% CO_2_. The mProx24 cells were plated onto 60 mm dishes and incubated with CsA at concentrations of 3, 10, and 30 μg·mL^−1^ for 12 and 24 h to determine the effect of 5‐ALA/sodium ferrous citrate (SFC) on cyclosporine‐induced renal injury. For antioxidant treatment, the cells were treated with combined 5‐ALA (1 mm) and SFC (0.5 mm). Individual experiments were repeated at least three times using various cell preparations.

### Transfection of mProx24 cells with HO‐1 siRNA

In the HO‐1‐siRNA experiment, the mProx24 cells were grown to 60–80% confluence in the culture medium and then were transfected with HO‐1‐siRNA 13 or control‐siRNA using an siRNA transfection reagent (Life Technologies, Grand Island, NY, USA). Later, the transfection solution was removed, and the cells were washed with PBS two times after transfection for 24 h.

### Measurement of mProx24 cell apoptosis by flow cytometry

The mProx24 cells were treated with CsA or CsA plus 5‐ALA/SFC and then were plated in six‐well plates. After incubation for 12 and 24 h, the cells were stained with FITC‐Annexin V (Biolegend, Tokyo, Japan) and propidium iodide (PI) and washed with PBS. They were then centrifuged at 270 ***g***. for 5 min. The cells were resuspended at a density of 1 × 10^6^ cells per mL with Annexin‐binding buffer (Biolegend). To every 100 μL of cell suspension, 5 μL of FITC‐Annexin V and 2 μL of PI were added. The cells were incubated at room temperature for 20 min. Then, 500 μL of 1× Annexin‐binding buffer was added, and the cells were analyzed immediately by flow cytometer (Gallios; Beckman Coulter, Tokyo, Japan).

### Assessment of markers for renal ROS

To assess the production of reactive oxygen species (ROS), mProx24 cells were incubated with 5/6‐chloromethyl‐2′,7′‐dichlorodihydrofluorescein diacetate, acetyl ester (CM‐H2DCFDA) (Invitrogen, Burlington, ON, Canada). Samples were analyzed using a flow cytometer (Gallios).

### Western blot analysis

For western blot analysis, the mProx24 cells were washed twice with cold PBS, and cell lysate was prepared using RIPA buffer (Wako, Osaka, Japan). The total proteins (40 μg) were separated on 10% SDS/PAGE, then transferred onto a polyvinylidene difluoride membrane (Bio‐Rad, Hercules, CA, USA). The membranes were blocked with 3% skim milk for 1 h at room temperature, then incubated with primary antibodies overnight at 4 °C. The proteins were extracted and blotted with antibodies to α‐tubulin (1 : 4000; Santa Cruz Biotechnology, Santa Cruz, CA, USA), HO‐1 (1 : 2000; Abcam, Cambridge, UK), Nrf2 (1 : 2000; Santa Cruz Biotechnology), p38, phosphoinositol‐p38 (p‐P38) (1 : 1000; Cell Signaling Technology, Beverly, MA, USA), Bcl‐2 (1 : 2000), caspase‐3 (1 : 2000), phosphoinositol‐Erk (p‐Erk)‐1/2, and p‐p‐Erk‐1/2 (1 : 1000; Cell Signaling Technology). Appropriate HRP‐conjugated secondary antibodies were applied for 1 h at room temperature. The proteins were detected with SuperSignal West Pico Chemiluminescent Substrate solution (Thermo Scientific, Rockford, IL, USA). Image Quant LAS 4000 (GE Healthcare, Little Chalfont, Buckinghamshire, UK) was used to measure the relative optical density of each specific band obtained, and α‐tubulin was used to normalize protein loading.

### Immunofluorescence analysis

Cells grown on coverslips were fixed with 4% PFA in PBS for 20 min and permeabilized with 0.5% Triton X‐100, then blocked with 1% BSA in PBS for 60 min at room temperature. Rabbit polyclonal anti‐Nrf2 (1 : 200; Santa Cruz Biotechnology) and rabbit polyclonal anti‐Bax (1 : 200; Santa Cruz Biotechnology) were incubated for 18 h at 4 °C. After three PBS washes, the cells were incubated with FITC‐conjugated rabbit immunoglobulin G secondary antibody (1 : 200, Santa Cruz Biotechnology) at room temperature for 1 h, then washed with PBS. To detect the nuclear expression of Nrf2, cell nuclei were stained with PI (0.1 g·mL^−1^) for 1 min. Sections were viewed under a fluorescence microscope (BX51; Olympus, Tokyo, Japan) using a fluorescein FITC and PE color filter, and the results were analyzed using image j software (https://imagej.nih.gov/ij/).

### Mitochondrial labeling

To label the mitochondria, mProx24 cells were loaded with 200 nm MitoTracker Green (M7514; Thermo Fisher Scientific K.K., Tokyo, Japan), which passively diffuses across the plasma membrane and accumulates in the mitochondria 16, at 37 °C for 30 min and then were washed three times. Cells on the coverslips were fixed with 4% PFA in PBS for 10 min at room temperature, rinsed with PBS, and then permeabilized with 0.2% Triton X‐100 for 5 min. The coverslips were observed under a fluorescence microscope (BX51).

### TUNEL staining

Apoptosis was detected by the TUNEL method using an *in situ* Apoptosis Detection Kit (Trevigen, Inc., Gaithersburg, MD, USA) according to the manufacturer's instructions. After 24 h of CsA treatment, the mProx24 cells were fixed in 4% PBS‐buffered paraformaldehyde for 30 min at room temperature and then permeabilized with 0.1% Triton X‐100 in sodium citrate for 2 min on ice. The cells were then incubated with 50 μL terminal deoxynucleotidyl transferase end‐labeling solution for 60 min at 37 °C in a darkened, humidified chamber and counterstained in PI staining solution for 5 min at room temperature. The stained cells were then washed one to two times with PBS and examined by fluorescence microscopy (BX51). The percentage of positively stained cells was calculated as the percentage of the apoptotic cells relative to the total number of cells.

### Statistical analysis

The mean ± SD was calculated for all the parameters in this study. Statistical significance was evaluated using Student's *t*‐test. Correlations were determined by Spearman's ranking. *P *<* *0.05 was considered statistically significant.

## Results

### Effects of 5‐ALA/SFC treatment on CsA‐induced apoptosis in mProx24 cells

**Figure 1:**
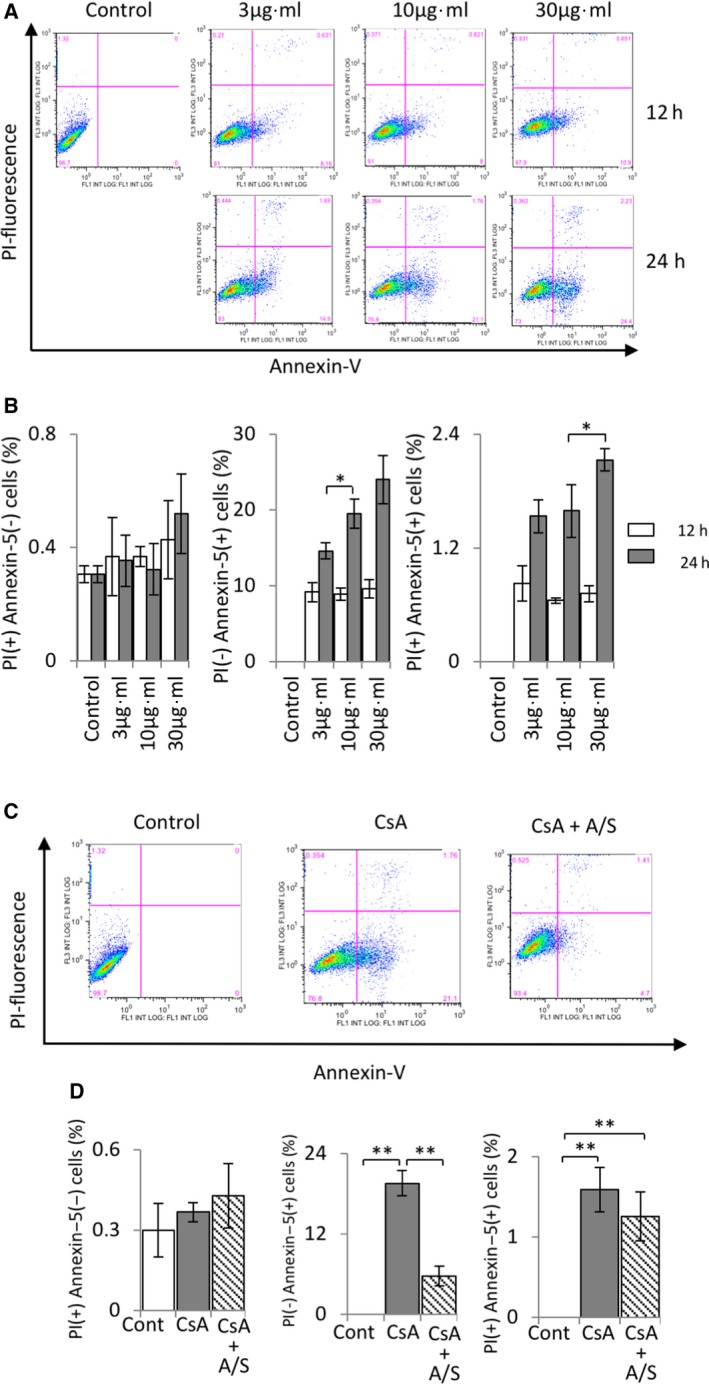
The effect of 5‐ALA/SFC on CsA‐induced apoptosis in mProx24 cells. (A) Apoptosis detected by flow cytometry; expression of Annexin V (+) PI (–) in mProx24 cells treated with CsA (3, 10, and 30 μg·mL^−1^) for 12 and 24 h. (B) Quantitative analysis of data from (A). (C) 5‐ALA/SFC suppressed CsA‐induced cell apoptosis. (D) Quantitative analysis of data from (D). The data represent mean ± SD. Statistical significance was measured by Student's *t*‐test. Correlations were determined by Spearman's ranking (*n* = 3; *P <* *0.05, **P <* *0.01).

To investigate the CsA dosage and time‐dependent apoptotic damage to the mProx24 cells, we applied CsA at the concentrations of 3, 10, and 30 μg·mL^−1^ for two different time periods (12 and 24 h). CsA‐induced apoptosis of mProx24 cells was detected by flow cytometry after staining with Annexin V and PI (Fig. 1A,B) at 12 h after CsA treatment. The Annexin V‐positive population increased from 0.01% to 10%. However, there were no significant differences among the groups receiving the three different concentrations. In contrast, at 24 h after CsA treatment, the Annexin V‐positive population increased from 0.01% to 23.5% in a dose‐dependent manner (Fig. 1A,B). Based on the findings above, in the next phase of our experiment we incubated the mProx24 cells with CsA (10 μg·mL^−1^) for 24 h, then applied 5‐ALA/SFC. As shown in Fig. 1C,D, 5‐ALA/SFC treatment decreased CsA‐induced apoptosis significantly.

### 5‐ALA/SFC upregulated HO‐1 and Nrf2 expression in CsA‐treated mProx24 cells

**Figure 2:**
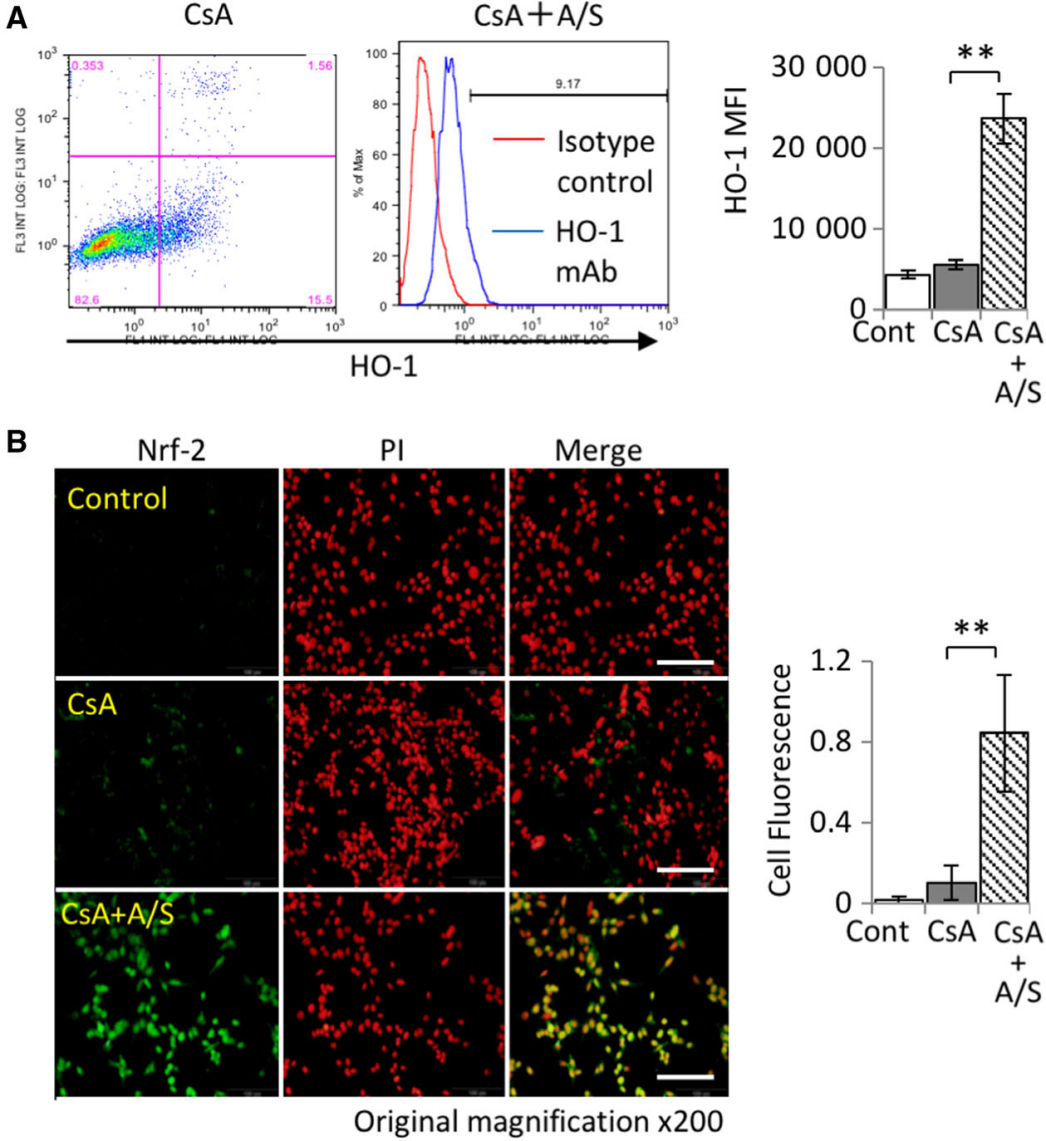
5‐ALA/SFC increased HO‐1 and Nrf2 expression in mProx24 cells. (A) Effect of 5‐ALA/SFC on HO‐1 production in kidney cells. HO‐1 expression increased on 5‐ALA and SFC exposure but did not change after CsA (30 μg·mL^−1^) treatment in mProx24 cells for 24 h (*n* = 3; **P <* *0.01). (B) Nrf2 expression (FITC) after 5‐ALA/SFC treatment for 24 h. Data are shown as the percentage of Nrf2‐positive cells (scale bar is 200 μm; *n* = 3; *P <* *0.05, **P <* *0.01). The data represent mean ± SD. Statistical significance was measured by Student's *t*‐test. Correlations were determined by Spearman's ranking.

Our findings thus far indicated that 5‐ALA/SFC‐induced HO‐1 expression exerted a critical cytoprotective effect against several harmful stimuli 8, 12, 13, 17, 18. To investigate this phenomenon further, we assessed the intracellular expression of HO‐1 by flow cytometric analysis. HO‐1 expression was significantly greater in CsA‐stimulated mProx24 cells with 5‐ALA/SFC treatment than in those without it (Fig. 2A). Furthermore, we performed immunocytofluorescence analysis to examine the expression of HO‐1 by Nrf2, one of the critical regulators of HO‐1 4, in mProx24 cells. After treatment with 5‐ALA/SFC, Nrf2 expression was significantly upregulated in the nucleus of mProx24 cells (Fig. 2B).

### HO‐1 silencing abrogated the inhibitory effect of 5‐ALA/SFC on apoptosis and oxidative stress in CsA‐stimulated mProx24 cells

**Figure 3:**
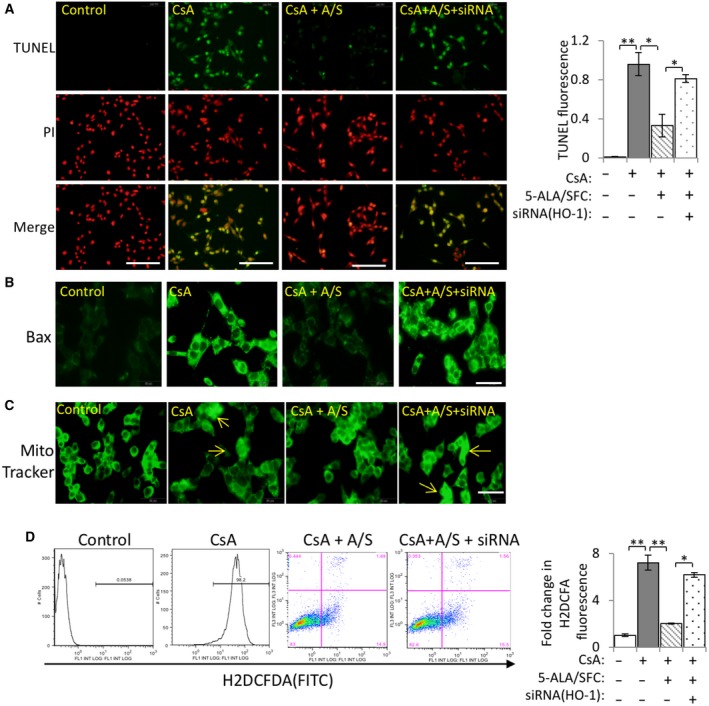
Effects of 5‐ALA/SFC on CsA‐induced oxidative stress and apoptosis. (A) Representative immunofluorescence microscopy images of TUNEL (scale bar: 100 μm) and quantitative data on TUNEL‐positive fluorescence. (B) Bax expression was translocated to the mitochondria (scale bar: 50 μm). (C) Representative fluorescence images from MitoTracker (mitochondria for green fluorescence) (scale bar: 50 μm). (D) Representative and quantitative data on the cell levels of CM‐H2DCFDA in four groups were assessed. Data are expressed as the mean ± SD. Statistical significance was measured by Student's *t*‐test. Correlations were determined by Spearman's ranking (*n* = 3; *P <* *0.05, **P <* *0.01).

To investigate the effect of 5‐ALA/SFC on HO‐1 expression in CsA‐induced apoptosis in mProx24 cells, the cells were transduced with HO‐1‐specific siRNA. In line with the data on Annexin V exposure obtained by flow cytometric analysis (Fig. 1A,B), CsA treatment demonstrated a significant increase in TUNEL‐positive programed cell death, whereas 5‐ALA/SFC treatment significantly suppressed the induction of TUNEL‐positive cells by CsA. In contrast, inhibition of HO‐1 by pretreatment with HO‐1‐specific siRNA resulted in a significant abrogation of 5‐ALA/SFC's suppressive effect by increasing the number of TUNEL‐positive (apoptotic) cells (Fig. 3A). Next, the effect of 5‐ALA/SFC‐mediated suppression of apoptosis on the expression of Bax, a pro‐apoptotic gene and a key mediator of mitochondrial damage during CsA‐induced apoptosis, was analyzed. As shown in Fig. 3B, CsA induced Bax expression, while 5‐ALA/SFC inhibited it. Furthermore, silencing HO‐1 expression suppressed the 5‐ALA/SFC‐mediated inhibitory effect on Bax. In addition, CsA induced the translocation of Bax to the mitochondria, resulting in the formation of mitochondrial aggregates (Fig. 3B); MitoTracker staining revealed that CsA treatment induced mitochondrial aggregation in the mProx24 cells, whereas 5‐ALA/SFC suppressed it. However, HO‐1 siRNA abrogated 5‐ALA/SFC's inhibitory function (Fig. 3C). An analysis of the effect of HO‐1 upregulation by 5‐ALA/SFC on CsA‐induced oxidative stress in mProx24 cells using fluorescent dye revealed that whereas CsA increased oxidative stress significantly in the cells, 5‐ALA/SFC suppressed ROS production. In contrast, HO‐1 siRNA abrogated the suppression of ROS production by 5‐ALA/SFC (Fig. 3D).

### 5‐ALA/SFC upregulated HO‐1‐related signaling molecules and anti‐apoptotic molecules and downregulated caspase activation in mProx24 cells treated with CsA, whereas HO‐1‐siRNA attenuated caspase activation

**Figure 4:**
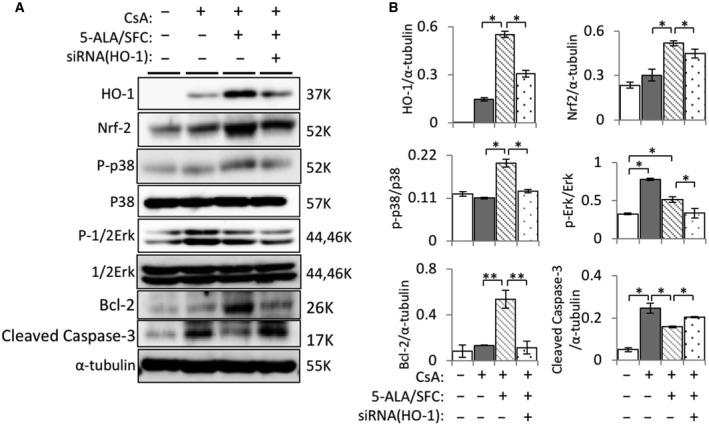
5‐ALA/SFC increased HO‐1 and Nrf2 expression via activation of MAPK signaling pathway in mProx24 cells. (A) After pretreatment with HO‐1 siRNA, representative bands of different groups showed HO‐1, Nrf2, p38, p‐p38, Erk‐1/2, p‐Erk‐1/2, Bcl‐2, and cleaved‐caspase‐3 proteins in mProx24 cells treated with 5‐ALA/SFC. (B) Quantitative densitometry was performed. Data are the mean ± SD. Statistical significance was measured by Student's *t*‐test. Correlations were determined by Spearman's ranking (*n* = 3; *P <* *0.05, **P <* *0.01).

To study the upstream signaling pathway involved in 5‐ALA/SFC‐induced HO‐1 expression, we examined the expression levels of Nrf2, p‐p38, p‐Erk‐1/2 and HO‐1 by western blot. As seen in Fig. 4, 5‐ALA/SFC upregulated HO‐1 and Nrf2 expression in CsA‐treated mProx24 cells, but silencing HO‐1 attenuated the increase in the expression of both molecules induced by 5‐ALA/SFC. Furthermore, 5‐ALA/SFC treatment showed increasing phosphorylation of p38 and Erk‐1/2 in CsA‐treated mProx24 cells and the control (Fig. 4A,B). Moreover, 5‐ALA/SFC attenuated the expression of pro‐apoptotic cleaved‐caspase‐3 and restored the expression of anti‐apoptotic Bcl‐2 protein in CsA‐treated mProx24 cells. In contrast, HO‐1‐siRNA transduction led to significantly reduced p‐p38 and p‐Erk‐1/2 expression, increased cleaved‐caspase‐3 levels, and decreased Bcl‐2 levels.

## Discussion

This may be the first report confirming the protective effect of 5‐ALA/SFC on CsA‐induced apoptosis *in vitro*. In the present study, we demonstrated the following five aspects of 5‐ALA/SFC activity on CsA‐stimulated mProx24 cells: (a) inhibition of CsA‐induced apoptosis; (b) induction of Bcl‐2 expression and reduction of Bax expression and caspase‐3 activation; (c) induction of HO‐1 and Nrf2 expression with p38 and Erk phosphorylation; (d) attenuation of mitochondrial morphological changes and ROS production; and (e) HO‐1 inhibition abrogating the therapeutic effect of 5‐ALA/SFC.

As we reported previously, 5‐ALA with SFC causes strong HO‐1 induction *in vivo* and *in vitro* as compared to 5‐ALA alone 8, 18. HO‐1 degrades heme into biliverdin, CO, and iron, and biliverdin is immediately reduced and turned into bilirubin by biliverdin reductase. As biliverdin/bilirubin and CO both have antioxidative functions, HO‐1 is deemed to be a promising drug target. These metabolites of heme protect against apoptosis, inflammation, and oxidative stress. Furthermore, HO‐1 has been reported to prevent kidney damage as it exhibits renal tropism and has antifibrosis effects in CsA‐induced nephrotoxicity. Therefore, we used 5‐ALA/SFC to increase expression of HO‐1 and its upstream and downstream factors to protect against CsA‐induced mProx24 cell injury.

Cyclosporin A remains the most widely used contemporary immunosuppressant in organ transplantation, but the long‐term use of CsA may lead to nephrotoxicity, a complex physiological process involving gene expression regulation and protein interactions. Therefore, it is always useful to develop adjuvants capable of increasing the efficacy of these drugs while reducing their potential toxicity. In line with previous reports, our data showed CsA‐induced apoptosis in mProx24 cells, a proximal tubular cell line 19.

Induction of apoptosis was mediated by the mitochondrial status, balance of pro‐apoptotic and anti‐apoptotic molecules, and caspase activation. CsA‐induced renal dysfunction and morphological changes were associated with mitochondrial damage in the kidneys 20. Furthermore, CsA induced apoptosis of the renal proximal tubule cells *in vitro* through mitochondrial‐dependent and mitochondrial‐independent pathways as well as partially through activation of caspase‐3 and oxidative stress 21. Our study also demonstrated that CsA‐induced apoptosis in mProx24 cells was accompanied by mitochondrial morphological changes, upregulation of pro‐apoptotic Bax proteins, downregulation of anti‐apoptotic Bcl‐2 protein, and caspase‐3 activation.

Several studies have shown that caspase inhibitors or knockout/knockdown of pro‐apoptosis‐related genes can prevent acute kidney injury 22, 23. In the present study, we used 5‐ALA/SFC to attenuate CsA‐induced nephrotoxicity in proximal tubular cells following our previous, successful use of 5‐ALA/SFC to attenuate cytotoxicity in macrophages and cardiomyocytes 8, 18. As expected, 5‐ALA/SFC suppressed CsA‐induced apoptosis and related apoptosis‐promoting events. To analyze the 5‐ALA/SFC activity inhibiting apoptosis, we examined HO‐1 expression induced by a variety of pro‐oxidant stimuli as an important stress response of the antioxidant enzyme 6. HO‐1 is a key enzyme in the antioxidant response of tubular cells, and upregulation of HO‐1 protects mitochondrial function 24. Previous reports demonstrated that HO‐1 is upregulated in response to oxidative stress in proximal tubular cells where it confers significant cytoprotective and anti‐inflammatory effects 5, 7. Our results indicated that HO‐1 was upregulated with 5‐ALA/AFC in mProx24 cells, suggesting that it might play a key role in the survival of 5‐ALA/SFC‐treated cells *in vitro*. To determine whether 5‐ALA/SFC enhanced HO‐1 attenuated CsA‐induced cytotoxicity in mProx24 cells, HO‐1 gene silencing was performed by HO‐1‐specific siRNA transduction. As expected, silencing of HO‐1 inhibited the cytoprotective effects of 5‐ALA/SFC.

The mechanism of HO‐1 regulation mainly includes hypoxia and inflammation signals, such as IL‐1, TNF‐α, Nrf2, and heme levels. Nrf2 is a redox‐sensitive alkaline leucine zipper transcription factor. The expression of phase II detoxification and antioxidant enzymes (including HO‐1) was induced by the Nrf2/Keap1 transcription factor system 4. The primary control of Nrf2 transcriptional activation induced by phase II genes depends on the subcellular distribution in response to oxidative or electrophilic forces. It is well known that HO‐1 gene regulation involves the regulation of Nrf2 and Keap1 and their binding in the cytosol by ARE. Our results demonstrated that 5‐ALA/SFC treatment increased the expression and nuclear translocation of Nrf2 in mProx24 cells, suggesting that Nrf2 mediates the induction of HO‐1 expression in 5‐ALA/SFC‐stimulated mProx24 cells. Our previous studies demonstrated that the MAPK pathway regulated Nrf2 expression and nuclear translocation in murine macrophage and cardiomyocyte cell lines 13, 25. In this study, 5‐ALA/SFC also upregulated the MAPK pathway (phosphorylation of p38 and Erk) in mProx24 cells, suggesting that Nrf2 expression induced by 5‐ALA/SFC in mProx24 cells might regulate this pathway.

Our series of studies and other reports demonstrated that the combination of 5‐ALA and SFC had an immunomodulatory property ameliorating several types of autoimmune and inflammatory disorders in animal models 8, 18, 26. In this study, 5‐ALA/SFC attenuated CsA‐induced renal tubular cell death *in vitro* probably through protecting the mitochondria and enhancing HO‐1 expression. The upregulation of HO‐1 may be involved in the increased expression of the Nrf2 and MAPK pathway in line with our previous studies 13, 25. Furthermore, we recently demonstrated the attenuation of 5‐ALA/SFC tubulointerstitial fibrosis and renal apoptosis in a murine chronic cyclosporine nephropathy model 12. The findings of these previous studies and the current data strongly suggest that 5‐ALA/SFC has the potential to prevent CsA‐induced nephrotoxicity/nephropathy, although further research on the molecular mechanisms underpinning the cytoprotective effects of the 5‐ALA/SFC‐HO‐1/Nrf2 axis against CsA‐induced nephrotoxicity is needed before 5‐ALA/SFC can be used clinically for the treatment of CsA‐induced nephrotoxicity.

In this study, silencing of HO‐1 modulates expression of Nrf2 and phosphorylation of p38 and Erk‐1/2. HO‐1 is an inducible enzyme that catalyzes the rate‐limiting step in the oxidative degradation of heme to free iron, biliverdin, and CO. 5‐ALA/SFC induces CO via HO‐1 induction, which is mediated by Nrf2 as reported previously 8, 14, 17, 18, 27. Furthermore, CO‐releasing molecules or CO gas induces HO‐1 expression via Nrf2 activation *in vitro* and *in vivo* 28, 29, 30. Therefore, suppression of HO‐1 expression may prevent CO release, thereby affecting 5‐ALA/SFC‐activated Nrf2 expression decline. Furthermore, 5‐ALA/SFC upregulates phosphorylation of p38 and Erk‐1/2 MAPK as described previously 8, 13, 18, 25. Consistent with previous studies 8, 18, 5‐ALA/SFC upregulates phosphorylation of p38; in contrast, CsA‐mediated phosphorylation of Erk‐1/2 is downregulated by 5‐ALA/SFC treatment. The mechanism underlying the downregulation of Erk‐1/2 by 5‐ALA/SFC remains unclear. However, consistent with a previous report 31 that CsA increased phosphorylation of Erk‐1/2 in a pig renal tubular epithelial cell line, our study also showed phosphorylation of Erk‐1/2 by CsA treatment in renal tubular epithelial cells. A possible explanation for the alteration in phosphorylation status of MAPKs by HO‐1 knockdown is the negative/positive feedback loop in MAPK and Nrf2/HO‐1 pathway. Several reports have demonstrated that CO modulates phosphorylation of Erk‐1/2 and p38 32, 33, 34. Therefore, suppression of HO‐1 expression may prevent CO release, affecting 5‐ALA/SFC‐mediated phosphorylation of p38 and Erk‐1/2. However, further analysis with a specific inhibitor is needed to identify the exact mechanism underlying the regulatory function of 5‐ALA/SFC on the expression of Nrf2 and phosphorylation of p38 and Erk‐1/2 in CsA‐exposed renal tubular epithelial cells.

In sum, this study provided new evidence of 5‐ALA/SFC's protective effects against CsA‐induced kidney injury via preservation of mitochondrial integrity, ROS production, and apoptosis induction via upregulation of HO‐1 expression *in vitro* using murine proximal tubular epithelial cells (mProx24 cells).

## Conflict of interest

The authors declare no conflict of interest.

## Author contributions

CL, MF, SZ, YI, HI, KT, MN, TT, PZ, and XKL conceived and designed the project; CL acquired the data; CL, MF, and XKL analyzed and interpreted the data; CL, MF, and XKL wrote the paper. All authors read and approved the final manuscript.
